# Gene copy number variations in the leukocyte genome of hepatocellular carcinoma patients with integrated hepatitis B virus DNA

**DOI:** 10.18632/oncotarget.6895

**Published:** 2016-01-12

**Authors:** Yanan Pang, Weixing Guo, Jiaqi Wang, Guixia Xu, Kai Cheng, Guangwen Cao, Mengchao Wu, Shuqun Cheng, Shanrong Liu

**Affiliations:** ^1^ Changhai Hospital, Second Military Medical University, Shanghai, China; ^2^ Eastern Hepatobiliary Surgery Hospital, Second Military Medical University, Shanghai, China; ^3^ Department of Epidemiology, Second Military Medical University, Shanghai, China

**Keywords:** HBV-HCC, CGH, integration, biomarker

## Abstract

Integration of hepatitis B virus (HBV) DNA into the human liver cell genome is believed to promote HBV-related carcinogenesis. This study aimed to quantify the integration of HBV DNA into the leukocyte genome in hepatocellular carcinoma (HCC) patients in order to identify potential biomarkers for HBV-related diseases. Whole-genome comparative genomic hybridization (CGH) chip array analyses were performed to screen gene copy number variations (CNV) in the leukocyte genome, and the results were confirmed by quantitative polymerase chain reaction (qPCR). The commonly detected regions included chromosome arms 19p, 5q, 1q and 15p, where 200 copy number gain events and 270 copy number loss events were noted. In particular, gains were observed in 5q35.3 (OR4F3) and 19p13.3 (OR4F17) in 90% of the samples. Successful homologous recombination of OR4F3 and the HBV P gene was demonstrated, and the amplification at 5q35.3 is potentially associated with the integration of HBV P gene into natural killer cells isolated from peripheral blood mononuclear cells (PBMCs). Receiver operating characteristic (ROC) curve analysis indicated that the combination of OR4F3 and OR4F17 a novel potential biomarker of HBV-related diseases.

## INTRODUCTION

Hepatocellular carcinoma (HCC), the third leading cause of cancer-related mortality worldwide [1], is associated with various etiological factors, including hepatitis B virus (HBV) or hepatitis C virus (HCV) infections, alcohol consumption, and dietary aflatoxin [2]. Among those, HBV is a particularly important factor in China [3]. Previous studies showed that HBV carriers have a 100-fold greater risk of HCC than non-carriers [4]. Moreover, HBV-HCC exhibits a propensity to metastasize shortly after diagnosis [5]. Although surveillance and surgical interventions have improved prognoses, a large proportion of HBV-positive HCC patients experience metastasis or postsurgical recurrence. Consequently, the five-year survival rate for HCC is only 30% to 40% [6].

The integration of HBV DNA into the human genome is of paramount importance for altering the function of endogenous genes or inducing chromosomal instability in HBV-HCC [7]. Studies have shown that HBV DNA integration into the hepatocyte genome occurs in 85% to 90% of HBV-HCC cases [8] and leads to multiple genomic aberrations [9]. Viral DNA integration disrupts the function of several genes important for normal cell growth and differentiation. In addition to point mutations, structural variations, and DNA methylation, the virus-associated aberrations include gene disruption or activation, formation of chimeric viral-human fusion genes, and DNA copy number variations (CNV) in some genes (e.g., *cyclin A2*), which cumulatively lead to hepatocyte carcinogenesis [10]. HBV DNA may also integrate into kidney and pancreas cells, indicating HBV integration is not liver cell-specific [11, 12].

The interaction between HBV and the immune system appears to be a crucial event during HBV-HCC development [13]. Numbers of immunocytes, including monocytes, are sharply decreased in HBV infected patients [14] and genes regulating immune function, such as HLA, IL-28B, STAT4 and MICA, are mutated by HBV [15]. These mutational events may disrupt immune clearance of HBV. Following clinically resolved infections, covalently closed circular (ccc) HBV DNA emerges not only in hepatocytes but also in leukocytes [16], which indicates that HBV infection may be responsible for leukocyte dysfunction in the peripheral blood [17]. Whether HBV can integrate into the leukocyte genome remains unknown, however.

In recent years, comparative genomic hybridization (CGH) array chips have become a useful tool in detecting and mapping gene CNV [18]. To provide an overview of CNV in the leukocyte genome of HBV-HCC patients, we employed CGH array chips to analyze peripheral blood mononuclear cells (PBMCs) from 10 HCC patients who were HBV carriers. Our study identified common gene gains and losses in the leukocyte genome of the HBV-HCC patients. We identified 14 immune-related genes. Of those, two genes coding for olfactory receptors, OR4F3 and OR4F17, exhibited increased copy numbers in approximately 90% of the samples. Thus HBV DNA does appear to integrate into the leukocyte genome, and the CNV correlated with HBV-related disease diagnoses and evaluations.

## RESULTS

### Genomic DNA copy number variations (CNV) in the leukocyte genome of HBV-HCC patients

**Figure 1:**
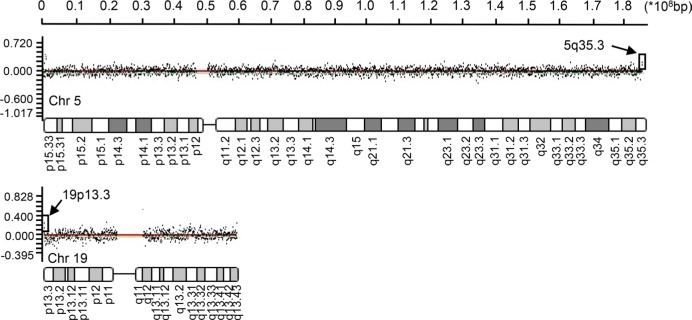
OR4F3 and OR4F17 amplifications A chromosome view of the CGH array results showing the terminal copy number gains on 5q35.3 and 19p13.3 including OR4F3 and OR4F17. Arrowheads indicate targets with upward-shifted log_2_ ratios. Chromosome (Chr).

Whole genome CGH array analyses of the peripheral blood leukocytes from 10 HBV-HCC patients was performed to study the CNV at a 70-kb average resolution (Supplementary Figure 1). The commonly detected regions included chromosome arms 19p, 5q, 1q, and 15p. The most frequent losses were observed at 1p34.1 (MSAT2), 8p21.3 (SEC22B), 9p12 (ZNF658B) 1q21.1 (PPLAL4E), 19p21.3 (HIST2H4A), 15q11.2 (BCL8), 8p23.1 (FAM66E), 16p11.2 (NF1P1) and 16p13 (MIR1977). Gains were observed at 1p11.2 (FAM72B), 1q21.2 (NBPF16), 15q11.2 (OR4M2) (GOLGA8C), 1p36.33 (LOC133331), 15q26.3 (POTEB), 16p11.2 (TP5STG3B), 19p13.3 (OR4F17), 8p27.2(SPAGE11A), 9p24.3 (FAM138C), 1p36 (SMN1) and 5q35.3 (OR4F3). 200 copy number gain events and 270 copy number loss events were noted. We analyzed the 82 most frequently mutated genes (Table 1), which included 14 immune-related genes with high CNV (Table 2) using the process presented in Supplementary Figure 2. Although multiple chromosome regions were affected, the most frequent gains (log_2_ ratio > 0.75) were observed in the 5q35.3 and 19p13.3 regions in the olfactory receptor genes in 90% of the samples (Figure 1, indicated by arrows). The observed olfactory receptor (OR) genes included two members: OR4F3 (chromosome5, 181367287-181368225) and OR4F17 (chromosome 19, 107152-111690), presented in Figure 1.

**Table 1:** Genomic gains and losses of genes in the leukocyte genome

Gene	Chromosome	Ref.seq	Start	End	Gene size(kb)	Train start	Train end	Frequency (%)	Gene Location	Gain or loss
FAM72B	1	NM_001100910	120279658	143991624	23711966	120640527	120657204	60	1p11.2	Gain
MAST2	1	NM_015112	46051093	46114083	62990	46041871	46274383	60	1p34.1	Loss
NBPF15	1	NM_173638	146330841	148150199	1819358	146827613	146862782	60	1q21.2	Gain
NBPF16	1	NM_001102663	146330841	148150199	1819358	146843980	146862891	60	1q21.2	Gain
NBPF20	1	NM_001037675	120279658	143991624	23711966	143326315	143540167	60	1q21.2	Gain
POTEB	15	NM_207355	18304999	20124999	1820000	19305252	19336667	50	15q26.3	Gain
PPIAL4A	1	NM_178230	146330841	148150199	1819358	147819626	147820286	60	1q21.1	Gain
PPIAL4E	1	NM_001144032	146330841	148150199	1819358	146910634	146911417	60	1q21.2	Gain
BCL8	15	NR_027992	18304660	20080292	1775632	19134810	19221496	40	15q11.2	Gain
FAM138A	1	NR_026818	20214	239271	219057	27220	28690	50	1p36.33	Gain
FAM138C	9	NR_026822	20214	239271	219057	27220	28690	50	9p24.3	Gain
FAM138F	19	NR_026820	20214	239271	219057	27220	28690	50	19p13.3	Gain
FAM66A	8	NR_026789	11874577	12618127	743550	12263898	12312881	40	8p23.1	Gain
FAM66E	8	NR_027424	7169866	7903147	733281	7849944	7903687	40	8p23.1	Gain
FLJ45445	7	NR_028324	20214	239271	219057	148015	153209	50	7q32.1	Loss
LOC 100132602	14	NR_028325	30634	765731	735097	313754	318443	60	14q11.1	Gain
LOC 100133331	1	NR_028327	30634	717252	686618	651002	655594	60	1p36.33	Gain
LOC 727924	15	NR_015416	18304660	20080292	1775632	19779395	19872450	40	15q11.2	Loss
*MIR1977	1	NR_031741	30634	717252	686618	556050	556128	60	MT	Loss
CXADRP2	15	NR_024387	18304999	20124999	1820000	19267798	19270255	50	15q11.2	Gain
GOLGA8C	15	NR_027411	18304999	20124999	1820000	19027687	19041040	50	15q11.2	Loss
LOC 646214	15	NR_027053	18304999	20124999	1820000	19185890	19194116	50	15q11.2	Gain
NF1P1	15	NR_028506	18304999	20124999	1820000	19386652	19399284	50	15q11.2	Gain
OR4N3P	15	NR_028067	18304999	20124999	1820000	19914825	19915757	50	15q11.2	Gain
ZNF658B	9	NR_027861	38751600	70210086	31458486	39433819	39437188	40	9p12	Gain
GOLGA6L6	15	NM_001145004	18304999	20124999	1820000	18997107	19007128	50	15q11.2	Loss
FAM90A7	8	NM_001136572	7244999	7804999	560000	7401070	7404644	40	8p23.1	Gain
DEFB103A	8	NM_018661	7244999	7804999	560000	7273825	7275092	40	8p23.1	Gain
DEFB103B	8	NM_001081551	7244999	7804999	560000	7273900	7275280	40	8p23.1	Gain
DEFB104A	8	NM_080389	7244999	7804999	560000	7315239	7320014	40	8p23.1	Gain
DEFB104B	8	NM_001040702	7244999	7804999	560000	7315239	7320014	40	8p23.1	Gain
DEFB105A	8	NM_152250	7244999	7804999	560000	7716939	7718770	40	8p23.1	Gain
DEFB105B	8	NM_001040703	7244999	7804999	560000	7716939	7718770	40	8p23.1	Gain
DEFB106A	8	NM_152251	7244999	7804999	560000	7327435	7331319	40	8p23.1	Gain
DEFB106B	8	NM_001040704	7244999	7804999	560000	7327435	7331319	40	8p23.1	Gain
DEFB107A	8	NM_001037668	7244999	7804999	560000	7706651	7710648	40	8p23.1	Gain
DEFB107B	8	NM_001040705	7244999	7804999	560000	7706651	7710648	40	8p23.1	Gain
SPAG11B	8	NM_058202	7244999	7804999	560000	7292685	7308602	40	8p23.1	Loss
OR4F16	1	NM_001005484	34999	524999	490000	58953	59871	40	1p36.33	Gain
OR4F17	19	NM_001005240	34999	174999	140000	61678	62596	90	19p13.3	Gain
OR4F29	1	NM_001005221	34999	524999	490000	357521	358460	40	1p36.33	Gain
OR4F3	5	NM_001005224	34999	524999	490000	357521	358460	90	5q35.3	Gain
OR4F5	1	NM_001005277	34999	524999	490000	357521	358460	40	1p36.33	Gain
OR4N4	15	NM_001005241	18304999	20124999	1820000	19883836	19884787	50	15q11.2	Gain
OR4M2	15	NM_001004719	18304999	20124999	1820000	19869939	19870881	50	15q11.2	Gain
SEC22B	1	NM_004892	120279658	143991624	23711966	143807763	143828279	60	1q21.1	Loss
BOLA1	1	NM_016074	146330841	148150199	1819358	148137778	148138971	60	1q21	Gain
FAM75A5	9	NM_001113541	38751600	70210086	31458486	41311106	41317364	40	9p12	Loss
FAM75A7	9	NM_015667	38751600	70210086	31458486	41311109	41317364	40	9q12	Loss
HIST2H2AA3	1	NM_003516	146330841	148150199	1819358	148089251	148089785	60	1q21.2	Gain
HIST2H2AA4	1	NM_001040874	146330841	148150199	1819358	148089251	148089785	60	1q21.2	Gain
HIST2H2AC	1	NM_003517	146330841	148150199	1819358	148125148	148125585	60	1q21.2	Gain
HIST2H3A	1	NM_001005464	146330841	148150199	1819358	148090804	148091311	60	1q21.2	Gain
HIST2H3C	1	NM_021059	146330841	148150199	1819358	148090804	148091311	60	1q21.2	Gain
HIST2H4A	1	NM_003548	146330841	148150199	1819358	148070844	148071240	60	1q21.2	Gain
HIST2H4B	1	NM_001034077	146330841	148150199	1819358	148070844	148071240	60	1q21.2	Gain
FAM66D	8	NR_027425	11874577	12618127	743550	12010699	12046107	40	8p23.1	Loss
GTF2H2D	5	NM_001042490	68914999	70664999	1750000	68891829	68924108	40	5q13.2	Loss
GTF2H2C	5	NM_001098728	68914999	70664999	1750000	68891806	68924485	40	5q13.2	Loss
GTF2H2B	5	NM_001098729	68914999	70664999	1750000	68891824	68924485	40	5q13.2	Loss
TP53TG3B	16	NM_001099687	32444999	32724999	280000	32592341	32596379	40	16p11.2	Loss
TP53TG3	16	NM_016212	32444999	32724999	280000	32592349	32595554	40	16p13	Loss
MSTP2	1	NR_027504	16834999	16974999	140000	16844655	16849501	40	1p36.2	Gain
LOC 100132287	1	NR_028322	34999	524999	490000	313754	318443	40	1p36.33	Gain
LCE3C	1	NM_178434	150822555	150852635	30080	150839761	150840186	60	1q21.3	Loss
NAIP	5	NM_022892	68888924	70682363	56632	70300065	70356697	50	5q13.2	Gain
SMN1	5	NM_022874	69386142	70704534	1318392	70256523	70284592	50	5q13.2	Gain
SMN2	5	NM_022877	69386142	70704534	1318392	69381105	69409177	40	5q13.2	Gain
DEFB109P1B	8	NR_003668	7169866	7903147	733281	7885347	7892453	30	8p23.1	Gain
LOC 653391	5	NR_029426	68888924	70682363	1793439	68713442	69917303	40	5q13.2	Gain
LOC 653501	9	NR_003528	38751600	70210086	31458486	39433813	39454526	30	9p13.1	Gain
BTNL8	5	NM_197975	180355922	45982	18034890	180348506	180366333	20	5q35.3	Loss
SPAG11A	8	NM_001081552	7153912	7982049	828137	7742811	7758729	60	8p27.2	Gain
ZNF705G	8	NM_001164457	7153912	7982049	828137	7202908	7207900	50	8p23.1	Gain
FAM86B2	8	NM_001137610	11874577	12618127	743550	12327496	12338223	50	8p23.1	Loss
FAM90A10	8	NM_001164447	7169866	7903147	733281	7663908	7666919	60	8p23.1	Loss
FAM90A13	8	NM_001164456	7169866	7903147	733281	7610374	7613384	60	8p23.1	Loss
FAM90A14	8	NM_001164452	7169866	7903147	733281	7101835	7104845	50	8p23.1	Loss
FAM90A18	8	NM_001164451	6891623	7903147	1011524	7618022	7621032	40	8p23.1	Loss
FAM90A19	8	NM_001164449	7153912	7982049	828137	7648613	7651623	40	8p23.1	Loss
FAM90A20	8	NM_001164453	6891623	7903147	1011524	7139945	7142955	40	8p23.1	Loss
FAM90A5	8	NM_001164455	6891623	7903147	1011524	7132323	7135333	30	8p23.1	Loss

* Ref.seq: The Reference Sequence (RefSeq) collection provides a comprehensive, integrated, non-redundant, well-annotated set of sequences, including genomic DNA, transcripts, and proteins. Frequency: Mutated frequency for genes in the 10 PBMC samples of CGH chips for HBV-HCC patients. *MIR1977 belongs to the MT (mitochondrial) genome.

**Table 2:** Copy number variations of immune-related genes in the leukocyte genome

Gene	Chromosome	Ref.seq	Start	End	Gene size (kb)	Train start	Train end	Frequency (%)	Gene Location	Gain or loss
DEFB103A	8	NM_018661	7244999	7804999	560000	7273825	7275092	40	8p23.1	Gain
DEFB103B	8	NM_001081551	7244999	7804999	560000	7273900	7275280	40	8p23.1	Gain
DEFB104A	8	NM_080389	7244999	7804999	560000	7315239	7320014	40	8p23.1	Gain
DEFB104B	8	NM_001040702	7244999	7804999	560000	7315239	7320014	40	8p23.1	Gain
DEFB105A	8	NM_152250	7244999	7804999	560000	7716939	7718770	40	8p23.1	Gain
DEFB105B	8	NM_001040703	7244999	7804999	560000	7716939	7718770	40	8p23.1	Gain
DEFB106A	8	NM_152251	7244999	7804999	560000	7327435	7331319	40	8p23.1	Gain
DEFB106B	8	NM_001040704	7244999	7804999	560000	7327435	7331319	40	8p23.1	Gain
DEFB107A	8	NM_001037668	7244999	7804999	560000	7706651	7710648	40	8p23.1	Gain
DEFB107B	8	NM_001040705	7244999	7804999	560000	7706651	7710648	40	8p23.1	Gain
DEFB109P1B	8	NR_003668	7169866	7903147	733281	7885347	7892453	30	8p23.1	Gain
BTNL8	5	NM_197975	180355922	45982	18034890	180348506	180366333	20	5q35.3	Loss
SPAG11A	8	NM_001081552	7153912	7982049	828137	7742811	7758729	60	8p27.2	Gain
LCE3C	1	NM_178434	150822555	150852635	150839761	150840186	353144	30	1p21.3	Loss

*Ref.seq: The Reference Sequence (RefSeq) collection provides a comprehensive, integrated, non-redundant, well-annotated set of sequences, including genomic DNA, transcripts, and proteins. Frequency: Mutated frequency for genes in the 10 PBMC samples of CGH chips for HBV-HCC patients.

We assessed OR4F3 and OR4F17 copy number levels by absolute qPCR in healthy controls (Con, *n* = 5), chronic hepatitis B patients (CHB, *n* = 10), hepatitis B virus-liver cirrhosis patients (HBV-LC, *n* = 10), and hepatitis B virus- HCC patients (HBV-HCC, *n* = 10). The results were consistent with the CGH array results. Compared with healthy controls, we observed up-regulation of OR4F3 and OR4F17 in HBV-related patients (*p*<0.05) (Supplementary Figure 3). The absolute amount of OR4F3 and OR4F17 DNA in the peripheral blood was highest in CHB patients, followed by HBV-LC patients, and lowest for HBV-HCC patients. All qPCR reaction efficiency values were between 1.95 and 1.99. The inter-assay variation in the qPCR efficiency was < 1%, confirming that the qPCR products were amplified at approximately the same rate.

### The HBV DNA integration into the leukocyte genome

**Figure 2:**
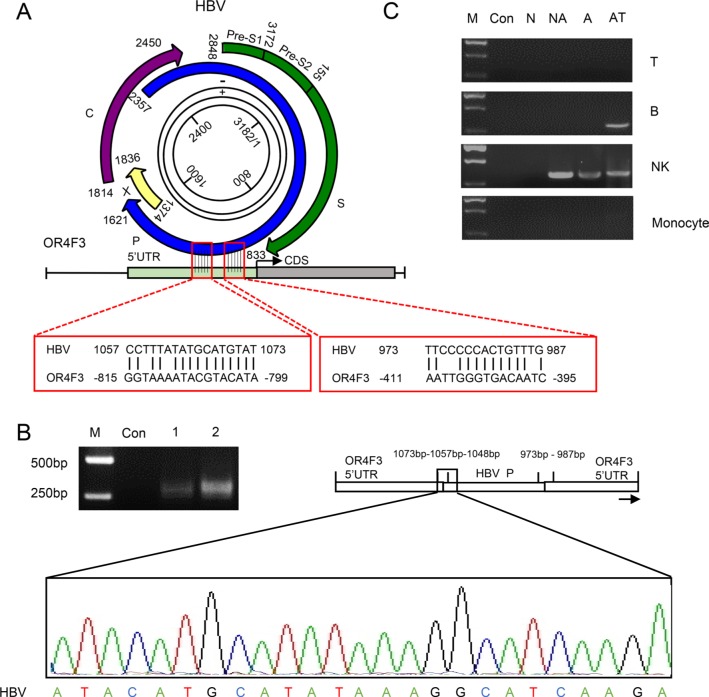
The homologous recombination of the OR4F3 5′UTR and HBV P gene **A.** A schematic alignment of OR4F3 and HBV is presented. The complementary sites between the OR4F3 gene and the HBV genome P gene are indicated by red frames. **B.** Compared with the control (Con), the agarose gel electrophoresis results show the OR4F3 and HBV chimeric fusion genes in the HBV-HCC samples. Sequencing results show one complementary site and a short HBV P gene sequence. The M lanes contain the DNA size markers (DL2000). The Con lane is healthy control, and lanes 1 and 2 are the HBV-HCC samples. UTR (untranslated region). **C.** The OR4F3 and HBV P gene chimeric fusion genes in the leukocyte genome are shown. Agarose gel electrophoresis showing the PCR amplification products for the healthy control (Con), non-HBV-HCC (N), non-active HBV-HCC (NA), active HBV-HCC (A), and antiviral therapy (AT) samples as well as marker (M). The chimeric fusion DNA fragments of P gene and OR4F3 were observed in the sorted NK cells of HBV- samples and B cells of samples from patient receiving antiviral therapy. T-lymphocyte cells (T cells); B-lymphocyte cells (B cells); Natural killer cells (NK cells).

Wing-Kin Sung's study found that the HBV DNA integration breakpoints appear to be associated with increased CNV [19]. To determine whether HBV DNA integrates into the leukocyte genome, a pairwise sequence alignment was performed between HBV and OR4F3 as a proof-of-principle study. Two complementary sites between these two sequences were identified (Figure 2A). In the human leukocyte genome, two sites at the OR4F3 5′ untranslated region (UTR) position (−395bp to −411bp and −799bp to −815bp) with an interval of 421 bp were complementary to the HBV P gene position sites (987bp to 973bp and 1073bp to 1057bp) with an interval of 101 bp. To test whether the 101 bp interval of the HBV P gene may interchange with the 421 bp interval that was located in the OR4F3 5′ UTR, PCR amplification was performed with a pair of exclusive primers, 5′–CTTGATGCCTTTATATGCATGTAT (HBV), 3′—CAACATGTGAGCTGCAAC CT (OR4F3, Supplementary Figure 4). As shown in Figure 2B, the specific recombination fragments, which were approximately 310 bp for HBV-HCC sample 1 and HBV-HCC sample 2, were derived from HBV in the OR4F3 5′ UTR. Additionally, the chimeric fusion genes (accession number: KT715476) were confirmed by sequencing the region between nucleotides 1048 and 1073 of the HBV P gene (Figure 2B, indicated by the black frames). This finding indicated the successful homologous recombination of the HBV P gene and the OR4F3 5′ UTR, thus confirming the integration of HBV DNA directly into the leukocyte genome.

Subsequently, CD3^+^ T (34.03%), CD19^+^ B (2.95%), CD3^−^CD56^+^ natural killer (NK) cells (26.89%) and CD14^+^ monocytes (1.89%) from PBMCs were sorted to determine the specific cell-lineages into which the HBV P gene integrated. The peripheral blood samples were obtained from healthy controls (Con, *n* = 3), non-HBV HCC patients (N, *n* = 3), non-active-HBV HCC patients (NA, *n* = 3), active-HBV HCC patients (A, *n* = 3), and from patients receiving antiviral therapy (AT, *n* = 3). As illustrated in Supplementary Figure 5, CD3^+^ T cells, CD19^+^ B cells, CD3^−^CD56^+^ natural killer (NK) cells and CD14^+^ monocytes were successfully isolated from PBMCs. Then, PCR analyses with the exclusive primers were used to identify the homologous recombination fragments of the P gene and OR4F3. As shown in Figure 2C, no specific P gene integration band was displayed in the four cell-lineages that were sorted from the healthy controls and the HBV-negative HCC samples. However, in the non-active HBV HCC sample lanes, active HBV HCC lanes, and samples from patient receiving antiviral therapy, bands that represented the chimeric fusion DNA fragments of P gene and OR4F3 were observed in the sorted NK cells. Moreover, bands that represented the chimeric fusion DNA fragments of P gene and OR4F3 were also observed in the sorted B cells of samples from patients receiving antiviral therapy. As shown in Figure 2C, the observed integration of the HBV P gene into the OR4F3 5 'UTR was cell-lineage specific.

### OR4F3 and OR4F17 are candidate biomarkers for HBV-related disease diagnoses

**Figure 3:**
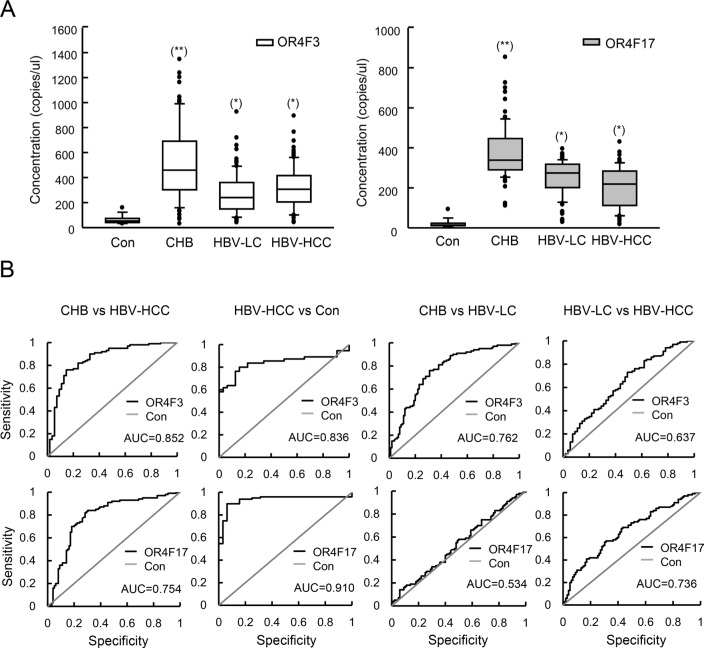
OR4F3 and OR4F17 as biomarkers for HBV-related diseases **A.** Quantification of OR4F3 and OR4F17 levels in healthy controls (*n* = 20), CHB patients (*n* = 100), HBV-LC patients (*n* = 100), and HBV-HCC patients (*n* = 100). The copy number levels were determined using absolute qPCR. Results are presented as copies per microliter. The unpaired t-test was performed to assess significance of differences between HBV-related groups and control groups. *P*-values of less than 0.05 were deemed to be significant. (*p < 0.05; **p < 0.005). Control (Con); chronic hepatitis B (CHB); hepatitis B virus-liver cirrhosis (HBV-LC); hepatitis B virus- hepatocellular carcinoma (HBV-HCC); OR4F3, olfactory receptor, family 4, subfamily F, member 3; OR4F17, olfactory receptor, family 4, subfamily F, member 17. **B.** ROC curves for the OR4F3 and OR4F17 genes between the CHB and HBV-HCC as well as HBV-HCC and normal are presented. OR4F3 yielded AUCs (areas under the ROC curve) of 0.852 for discriminating HBV-HCC from CHB and 0.836 for discriminating HBV-HCC from normal. OR4F17 yielded AUCs of 0.754 for discriminating HBV-HCC from CHB and 0.910 for discriminating HBV-HCC from normal. The ROC curves between CHB and HBV-LC as well as HBV-HCC and HBV-LC are presented. OR4F3 yielded AUCs of 0.762 for discriminating HBV-LC from CHB and 0.637 for discriminating HBV-HCC from HBV-LC. OR4F17 yielded AUCs of 0.534 for discriminating HBV-LC from CHB and 0.736 for discriminating HBV-HCC from HBV-LC. Control (Con).

We collected 320 samples of DNA from PBMCs from chronic hepatitis B (CHB), hepatitis B virus-liver cirrhosis (HBV-LC), HBV-HCC patients, and normal subjects (Supplementary Figure 6). As illustrated in Figure 3A, the absolute amounts of OR4F3 and OR4F17 approximately 9-fold and 16-fold higher in the CHB samples (*P* < 0.005), respectively, and 5-fold and 13-fold higher in the HBV-LC and HBV-HCC samples (*P* < 0.05), respectively, relative to healthy controls.

**Figure 4:**
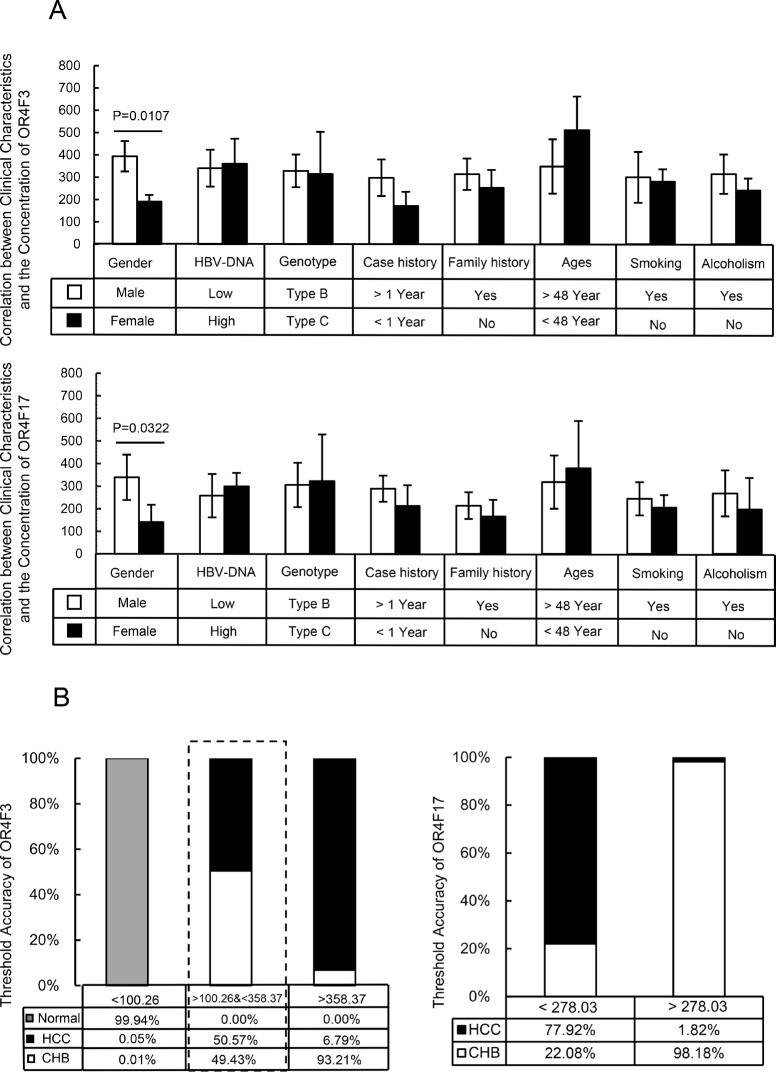
Clinical analysis of the samples and the threshold accuracy of OR4F3 and OR4F17 **A.** Correlation between clinical characteristics and the concentration of OR4F3 and OR4F17. According to the imaging and histological examination of liver, the patients were grouped into 100 CHB patients, 100 HBV-LC patients and 100 HBV-HCC patients. The concentration of OR gene did not correlate with the HBV-DNA level, genotype, case history, family history, ages, smoking, or alcoholism (*P*>0.05); however, the concentrations of OR4F3 and OR4F17 were varied between males and females (*P* = 0.0107 and *P* = 0.0322, respectively). Units: copies/ul. **B.** Threshold accuracy of OR4F3 and OR4F17. The threshold accuracy for OR4F3 and OR4F17. The cut-off value unit was copies/ul.

Subsequently, we evaluated whether OR4F3 and OR4F17 copy number could be used as diagnostic biomarkers for HBV-related diseases. ROC curve analyses were performed in pooled data sets. As shown in Figure 3B, we observed that OR4F3 copy number variation could distinguish CHB from HBV-HCC with an area under curves (AUC) of 0.852 (95% confidence interval (CI), 0.829-0.895) as well as HBV-HCC from healthy controls (Con) with an AUC of 0.836 (95% CI, 0.824-0.872). OR4F17 was also a potential biomarker for discriminating CHB from HBV-HCC with an AUC of 0.754 (95% CI, 0.715-0.803) as well as HBV-HCC from healthy controls (Con) with an AUC of 0.910 (95% CI, 0.894-0.936). However, OR4F3 could not discriminate CHB from HBV-LC with AUC of 0.762 or HBV-HCC from HBV-LC with AUC of 0.637, (95% CI not shown). OR4F17 was also not a potential biomarker for discriminating CHB from HBV-LC with AUC of 0.534 or for HBV-HCC from healthy controls with AUC of 0.736 (95% CI not shown). Furthermore, as shown in Figure 4A, the concentration of the OR genes did not correlate with the HBV-DNA level, genotype, case history, family history, age (group defined on the median of ages, Table 3), smoking, or alcoholism (*P* > 0.05). However, the concentrations of OR4F3 and OR4F17 were significantly different between males and females (*P* < 0.05).

**Table 3:** Observed clinical characteristics of CHB, HBV-LC and HBV-HCC patients

Copy number(/ml)	OR3<15.66			15.66<OR3<56.08			OR3>56.08		
Diagnosis	CHB	HBV-LC	HBV-HCC	CHB	HBV-LC	HBV-HCC	CHB	HBV-LC	HBV-HCC
**Number**	1	6	4	24	62	46	75	32	50
**Age (yrs)**	40	48±6.9	44±4	41±11.8	51±8.6	47±10.6	43±13.4	50±7.9	55±9.4
**Gender**									
**male**	1	6	3	16	48	26	48	26	28
**female**	0	0	1	8	14	20	27	5	22
**Biochemistry(IU/L)**									
**ALT**	14	40.0-44.0	9.0-12.0	25.5-137.0	37.8-139.8	18.5-40.9	33.3-147.8	37-158	17.5-38.6
**AST**	21	35.0-59.0	9.0-12.0	26.3-58.0	44.3-123.8	22.0-44.5	41.5-89.8	56-212	18.0-38.5
**AFP**	1.3	1.1-6.7	6.8-1629.7	8.0-127.9	1.1-7.1	2.9-32380.8	3.1-14.3	1.0-7.3	3.9-41283.8
**Tumor Sizes(cm)**	\	\	1.9-8.6*	\	\	1.2-11.5*	\	\	1.1-15.5*

Data are presented as mean +/− standard deviation.

* *P* <0.05.

Abbreviations: ALT, alanine aminotransferase; AST, aspartate aminotransferase; AFP, alpha-fetoprotein; yrs, years; HBV, hepatitis B virus; CHB, chronic hepatitis B; HBV-liver cirrhosis (HBV-LC); HBV-hepatocellular carcinoma (HBV-HCC).

## DISCUSSION

In this study, we identified multiple copy number alterations to the leukocyte genome in HBV-HCC patients. Of these, gains in two genes, OR4F3 and OR4F17, with a CNV frequency greater than 90% were noted at chromosome loci 5q35.3 and 19p13.3, respectively. Moreover, in combination OR4F3 and OR4F17 may provide a new biomarker for HBV-related disease diagnoses.

A specific fragment of the HBV genome, the P gene, was commonly integrated into the leukocyte genome upstream of OR4F3. Although the HBV cccDNA was observed in the cytoplasm of leukocytes, the fact that its integration is widespread in NK and B cells from patients receiving antiviral therapy has not been reported previously [20]. HBV DNA integration into the hepatocyte genome is a major mechanism underlying the etiologic role of HBV in HBV-HCC development [21]. A number of reports have indicated that HBx is one of the viral ORFs most commonly integrated into the hepatocyte genome, and its sequence variants play a crucial role in the progression of HBV-HCC [22]. HBx acts via the HBx-P3K-Akt-Bad pathway to inhibit caspase 3 function, thereby preventing p53-induced apoptosis and mediating induction of H-*ras* oncogene via phosphatidylinositol-3 kinase and Akt pathways [23]. Additionally, integration of the P region of HBV into the hepatocyte genome promotes genetic instability through mechanisms that include p53-mediated repression of gene transcription [24]. HBV integration also leads to elevated expression of several other cancer-related genes, including TERT, MLL4 and CCNE1 [25]. The dysregulation of numerous signaling pathways, including the MAPK, P53, sex steroid, Wnt/*β*-catenin, transforming growth factor β (TGFβ), PI3K/AKT, cytokine, IKK/NF-kB, and Hedgehog (Hh) pathways, has been observed in patients, and these changes are closely related with HBV-HCC development [26].

Our study revealed that genes exhibiting multiple copy number alterations, as detected using CGH array chips, may be associated with HBV DNA integration into the leukocyte genome. Alterations in chromosomal stability and changes in copy numbers are characteristic of HBV integrated events in HCC. The variation in copy numbers located within or near the integration sites suggests HBV DNA integration induces chromosomal instability. Jiang et al. previously showed that HBV DNA frequently integrates into the human genome, causing diverse changes, including DNA copy number variations, chimeric viral-human transcript fusions, and transcriptional activation [7]. Additional studies exploring possible tumorigenic roles of these genes with copy number changes would be of interest. Moreover, the underlying mechanisms of OR genes in leukocyte dysfunction, including weakened immune surveillance and HBV- HCC development, and the novel functions of HBV DNA integrated into the leukocyte genome provide interesting areas for future study.

Surprisingly, OR4F3 and OR4F17 copy numbers in the leukocyte genome serve as noninvasive biomarkers for diagnosing HBV-related diseases. We found that when the OR4F3 copy number is lower than 100.26 copies/μL, the probability that the samples were healthy controls was 99.94%, 0.01% for CHB, and 0.05% for HBV-HCC. When the OR4F3 copy number was higher than 358.37 copies/μL, the probability that the samples were from CHB patients, HBV-HCC patients or healthy controls was 93.21%, 6.79% and 0%, respectively. However, when the copy numbers were between 100.26 and 358.37 copies/μL, the probability that the samples were from CHB patients or HBV-HCC patients was 49.43% and 50.57%, respectively. Within the range of 100.26 to 358.37 copies/μL, these samples could be distinguished based on OR4F17. When the OR4F17 copy number was less than 278.03 copies/μL, the probability that the samples were from CHB or HBV-HCC patients was 22.08% and 77.92%, respectively. When the OR4F17copy number was greater than 278.03 copies/μL, the probability that the samples were from CHB or HBV-HCC patients was 98.18% and 1.82%, respectively (Figure 4B). However, these biomarkers could not distinguish HBV-LC from HBV-HCC. Given that liver cirrhosis development is the closest step to HBV-HCC, we hypothesize that the genomic aberrations in the HBV-LC and HBV-HCC patients were approximately the same. However, this hypothesis needs to be tested.

In summary, our results demonstrate that both integration of the HBV P gene into the 5′ UTR of OR4F3 genes and the copy numbers of OR genes were significantly increased within the leukocyte genome of HBV-related patients. Thanks to their reasonable sensitivity and specificity values for HBV-related diseases, it appears that OR4F3 and OR4F17 could potentially serve as diagnostic biomarkers. Several studies have also demonstrated that olfactory receptors possess oncogenic potential and can induce tumor formation when overexpressed in cells [27]. However, studies on the oncogenic potential of OR genes in the development of HBV-HCC have not been performed. Based on our present results, we are currently investigating the primary functions of OR genes duing the development and progression of HBV-HCC.

## MATERIALS AND METHODS

### Clinical samples

We performed clinical CGH array testing on 10 PBMCs samples from HBV-HCC patients from Eastern Hepatobiliary Surgery Hospital, Shanghai. HBV-HCC was diagnosed by the elevation of alpha-fetoprotein (AFP) (>400ng/mL) combined with at least one positive iconography examination result, including computed tomography (CT) and magnetic resonance imaging (MRI). The pathologic data of these 10 patients are summarized in Supplementary Table 1. They had pathological stage III (*n* = 7), pathological stage IV (*n* = 2), and pathological stage II (*n* = 1) according to the Edmondson-Steiner grading system. Four patients (40%) had tumor size less than 5 cm while one patient (10%) had tumor size of 11 cm. The rest (50%) had tumor sizes between 5 and 10 cm. These patients' ages ranged from 35-68 years with a median age of 43. They all had Child-Pugh class A liver function and were positive for HBV antigen (HBV-Ag). Their AFP levels ranged from 12-12430 ug/L. The ethics committee of the Eastern Hepatobiliary Surgery Hospital, Second Military Medical University (Shanghai, China), approved this study. Written informed consent was obtained from the patients according to the regulations of the committee.

### CGH array chips

For the CGH array experiments, 1 μg of purified patient DNA and control DNA were digested with 10U Alui and 10U RsaI and differentially labeled with cyanine-5 (Cy5) and cyanine-3 (Cy3) fluorescent dyes (PerkinElmer, Waltham, Massachusetts, USA) by random priming with the Bioprime CGH Array genomic labeling module (Invitrogen, Carlsbad, California, USA). Hybridization was performed for at least 20 h at 65°C in a rotating microarray hybridization chamber and then samples were washed according to the manufacturer's protocols (Agilent Technologies, Santa Clara, California, USA). The slide hybridization results were scanned into image files using a GenePix 4000B microarray scanner (Molecular Devices, Sunnyvale, California, USA). Then, Agilent's Feature Extraction v9.1 was used to quantify the array features, and the CGH analytics software was used to analyze the text file outputs for relative copy number changes. Common copy number variations were assessed by comparing these sample results with CNV databases and parental CGH patterns, when available.

### Microarray data analysis

Microarray data were analyzed with BRB-array tools. The Agilent Genomic Workbench was used to calculate the log_2_ ratios for every probe and to identify any genomic aberrations. The mean log_2_ ratios of all the probes in the chromosome regions between 0.25 and 0.75 were classified as genomic gains, > 0.75 was classified as high-level DNA amplification, < 0.25 was classified as homozygous losses, and < −0.75 was classified as homozygous deletions.

### Sample collection

Peripheral blood samples collected from 320 subjects were obtained from Eastern Hepatobiliary Hospital, Shanghai. According to imaging and histological examination of liver, patients were grouped into 100 CHB patients, 100 HBV-LC patients and 100 HBV-HCC patients. Twenty healthy control samples were obtained from the Physical Examination Center of Eastern Hepatobiliary Hospital. Five milliliters (mL) of peripheral blood was collected in K_3_-EDTA containing tubes, and PBMCs were isolated with 75% Ficoll Hypaque. PBMCs were distributed into aliquots and stored at −80°C until use.

The clinical characteristics of patients and controls, including age, gender etc., were investigated at the time of blood collection. The following laboratory parameters were obtained for each participant: aspartate aminotransferase (AST), alanine aminotransferase (ALT), and AFP. The main clinical characteristics of patients are summarized in Table 3.

Patients were divided into three main groups: (1) Group I-the CHB group (65 males and 35 females). CHB was diagnosed based on HBV-Ag seropositivity and continuously elevated ALT. Their ages ranged from 40-57 years with a median age of 48. The degree of inflammation of the CHB patients was between G1 and G2 and the fibrosis staging was between S0 and S2. (2) Group II-the HBV-LC group (81 males and 19 females). HBV-LC was diagnosed on the basis of pathology biopsy. Their ages ranged from 42-59 years with a median age of 51. The fibrosis staging of the HBV-LC patients was between S3 and S4. (3) Group III-the HBV-HCC group (57 males and 43 females). HBV-HCC was diagnosed by the elevation of AFP (>400ng/mL) combined with at least one positive iconography examination results, including CT and MRI. Their ages ranged from 37-65 years with a median age of 50. Twenty-three patients (23%) had tumor size less than 3 cm while sixteen patients (16%) had tumor size greater than 10 cm. The rest (61%) had tumor size between 3 and 10 cm. The fibrosis staging of the HBV-LC and HBV-HCC patients was between S3 and S4. All had Child-Pugh class A liver function. In addition, the patients with CHB, HBV-LC, and HBV-HCC were HBV-Ag positive in the serum and had not undergone anti-viral treatment.

### DNA purification

The genomic DNA of the PBMCs was extracted with a D70K PureGene DNA isolation kit (Qiagen/Gentra, Dusseldorf, Germany) according to the manufacturer's protocols. The genomic DNA was eluted in a final volume of 50 μL sterile water and was stored at −20°C until use.

### Sequences homology analysis

We performed pairwise sequence alignment analyses between the HBV and OR4F3 sequences using the basic local alignment search tool (BLAST, http://www.ncbi.nlm.nih.gov/).

### Construction of standard curves for qPCR

The partial OR4F3 and OR4F17 gene sequence were amplified by PCR. Amplicon sizes were verified by agarose gel electrophoresis and compared to a 2000-bp DNA ladder (Invitrogen, Burlington, Ontario, Canada). PCR fragments were cloned using the pMD^TM^ 18-T Vector Cloning kit, according to the manufacturer's manual (Takara). Plasmids were extracted using the DirectPrep 96 miniprep plasmid extraction kit (Qiagen, Mississauga, Ontario, Canada), and cloned fragments were verified by PCR and agarose gel electrophoresis. The plasmid copy number was quantified by spectrophotometry using a NanoDrop ND-1000 spectrophotometer (NanoDrop Technologies), and calculation of the gene copy numbers was performed according to the molar mass derived from the plasmid and amplicon sequences. A ten-fold dilution series over five points was prepared from 10^6^ copies/ul. The standard curves were constructed by plotting Cp values against the logarithmic concentration of the starting OR4F3 and OR4F17 with different concentrations. The amount of an unknown sample was quantified by interpolating the Cp values in the standard curves.

### Quantitative PCR

We used quantitative PCR (qPCR) to detect the copy number gains and losses in the genomic material to validate the representative CGH array results. qPCR was conducted in duplicate on a LightCycler 480II (Roche, Basel, Switzerland). Each 20 μL reaction consisted of 10 μL SYBR Premix DimerEraser^TM^ (Takara, Dalian, China), 250 nmol of each primer, 5 ng DNA sample, and 9 μL RNase-free water (Takara, Dalian, China). qPCR was conducted at 95°C for 30 s followed by 40 cycles at 95°C for 5 s and 72°C for 10 s. Melting curve analysis was performed to confirm the specificity of the PCR products.

### T cell, B cell, NK cell and monocyte purification with flow cytometry sorting

PBMCs were stained with CD3-APC, CD19-APC, CD56-PE, and CD14-APC antibodies (eBioscience, State of California, USA) at 4°C for 30 minutes [28]. Isotype controls with irrelevant specificities were included as negative controls. Human TruStain FcX™ (Fc Receptor Blocking Solution, Biolegend, San Diego, California, USA) was used to block Fc-receptors. Cells were washed three times with sterilized phosphate-buffered saline (PBS). CD3^+^ T cells, CD19^+^ B cells, CD3^−^CD56^+^ NK cells, and CD14^+^ monocytes were gated and isolated with a MoFlo XDP high-speed flow cytometry sorter (Beckman, Brea, California, USA). The purified cells were collected for CNV analysis of the OR gene.

### Statistical analysis

The distribution of the normalized OR4F3 and OR4F17 genes was characterized by their mean values and standard deviations. One-way analysis of variance (ANOVA) was used to compare the log_2_-transformed Cp values of the candidate reference genes among the groups. Student *t*-tests were used for the qPCR data to assess differences in CNV of the OR genes and the HBV-related samples. *P*-values < 0.05 were considered significant. Using the Statistical Package for Social Sciences (SPSS), the data were summarized in ROC curves to visualize the efficacy of OR4F3 and OR4F17 as biomarkers for HBV-related diseases. In these curves, the sensitivity (true positives) was plotted on the Y-axis, and the specificity (false positives) on the X-axis. All of the observed values were considered as arbitrary cutoff values. The AUC values were calculated as a measure for the discriminative efficacy of the tested biomarkers.

## SUPPLEMENTARY DATA FIGURES AND TABLE


