# Supplementary material for: Gene copy number variations in the leukocyte genome of hepatocellular carcinoma patients with integrated hepatitis B virus DNA
Source: Oncotarget. 2016 Jan 12;7(7):8006–18. doi: 10.18632/oncotarget.6895 (PMC4884971; doi:10.18632/oncotarget.6895)
Supplement: Supplementary file 1 [file oncotarget-07-8006-s001.pdf]

SUPPLEMENTARY DATA

Comparative genomic hybridization (CGH) chip array analyses were performed using peripheral blood mononuclear cells (PBMCs) to screen gene copy number variations (CNV) in the leukocyte genome. The microarray data were analyzed with BRB-array tools. Aligent Genomic Workbench was used to calculate the $\log_2$ ratios for every probe and to identify any genomic aberrations [1]. The mean log2 ratios of all the probes in the chromosome regions between 0.25 and 0.75 were classified as genomic gains, > 0.75 was classified as high-level DNA amplification, < 0.25 was classified as homozygous losses, and < -0.75 was classified as homozygous deletions [2]. In total, 200 copy number gain events and 270 copy number loss events were noted.

REFERENCES

1. Fischer U et al. Gene amplification during differentiation of mammalian neural stem cells in vitro and in vivo. Oncotarget. 2015 Mar 30; 6:7023–39.
2. Linuma H. Exosomal microRNA in serum is a novel biomarker of recurrence in human colorectal cancer. Br J Cancer. 2015 Jun 9. doi: 10.1038/bjc.2015.201.

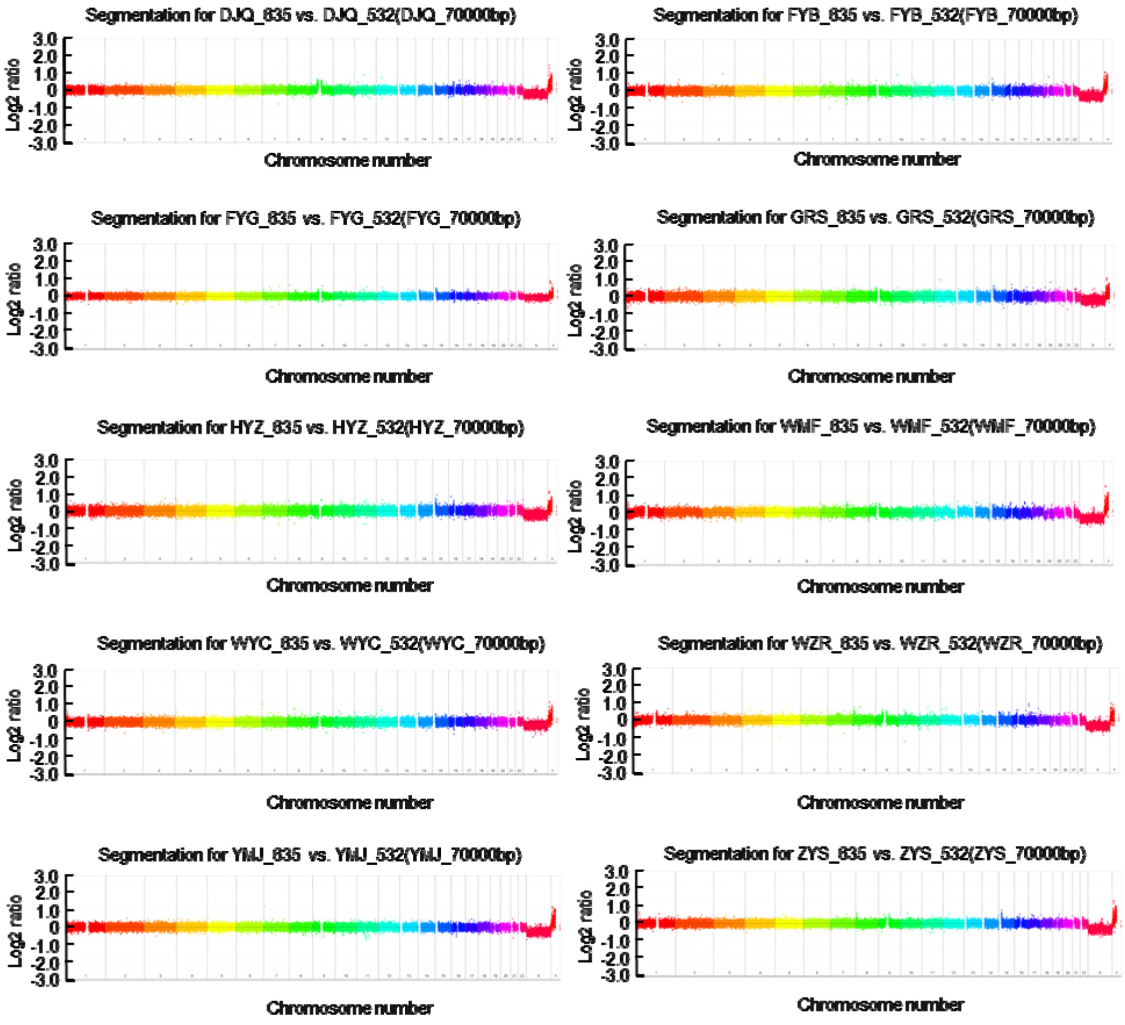

Supplementary Figure S1: A whole-genome CGH array analysis of the initial 10 samples. Log_2 ratio on the vertical axis was plotted against the genome position on the horizontal axis. Each point represents the average florescence intensity of all of the oligos that were contained within a 70-kb window, with the midpoint of the window used as the genome position. The dashed vertical lines denote breakpoints that represent the automated segmentation.

The flow-process diagram is presented in supplementary Figure 2. The most frequent gains (log_2 ratio > 0.75) were observed in the 5q35.3 and 19p13.3 regions and were olfactory receptor gene members in 90% of the samples (Figure 1A, indicated by arrows); therefore, these genes were further evaluated.

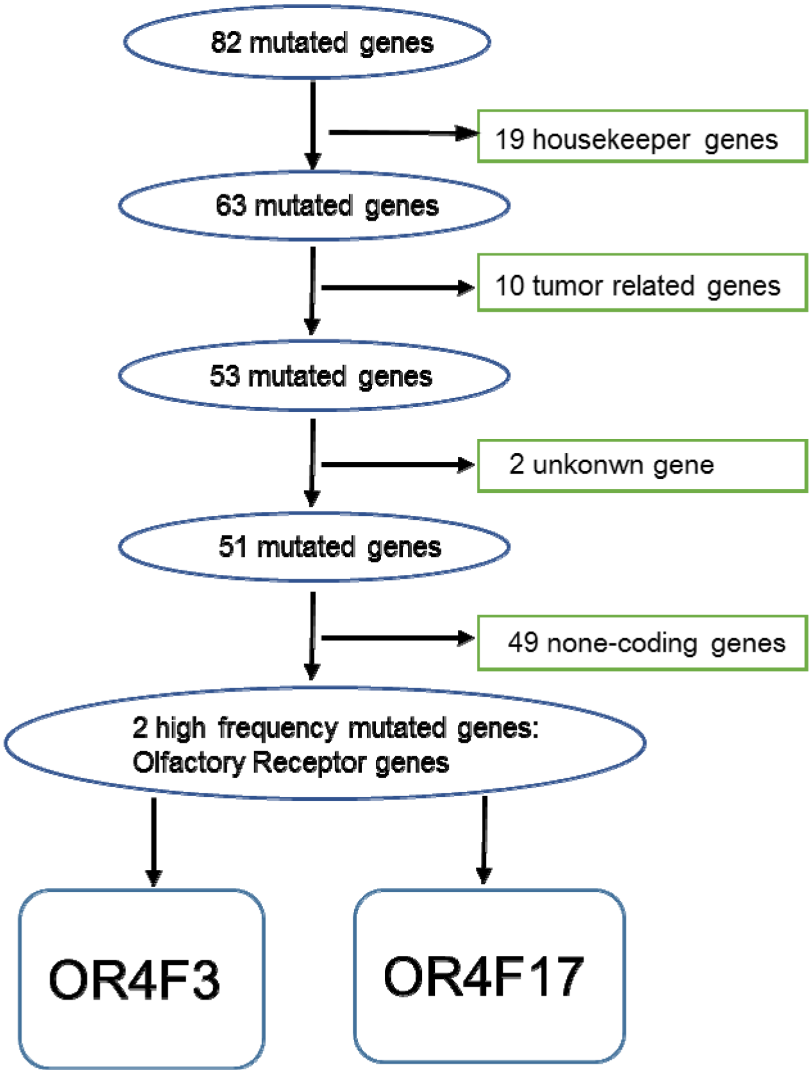

Supplementary Figure S2: Study profile. Screening steps for the target genes. OR4F3, olfactory receptor, family 4, subfamily F, member 3; OR4F17, olfactory receptor, family 4, subfamily F, member 17.

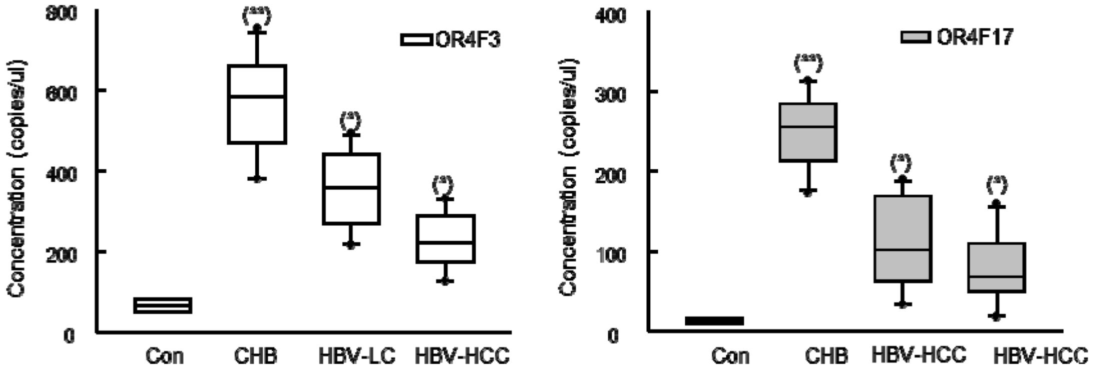

Supplementary Figure S3: Quantification of OR4F3 and OR4F17 levels in healthy controls (*n* = 5), CHB patients (*n* = 10), HBV-LC patients (*n* = 10) and HBV-HCC patients (*n* = 10). Results are presented as copies per microliter. The unpaired *t*-test was performed to assess significance of differences between HBV-related groups and control groups. P-values of less than 0.05 were deemed to be significant. (*$p < 0.05$; **$p < 0.005$). Control (Con); chronic hepatitis B (CHB); hepatitis B virus-liver cirrhosis (HBV-LC); hepatitis B virus-hepatocellular carcinoma (HBV-HCC); OR4F3, olfactory receptor, family 4, subfamily F, member 3; OR4F17, olfactory receptor, family 4, subfamily F, member 17.

PCR amplification was performed with a pair of exclusive primers, 5'–CTTGATGCCTTTATATGCATGTAT (HBV), 3'—CAACATGTGAGCTGCAAC CT(OR4F3).

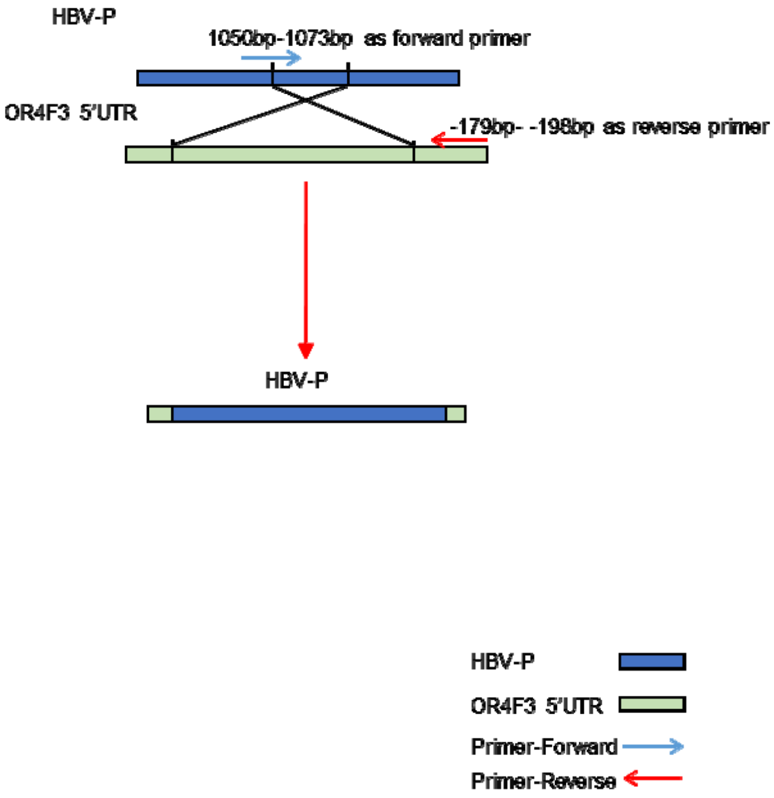

Supplementary Figure S4: Schematic diagram of the exclusive primer and the homologous recombination of the OR4F3 5' UTR and the HBV P gene. UTR (untranslated region).

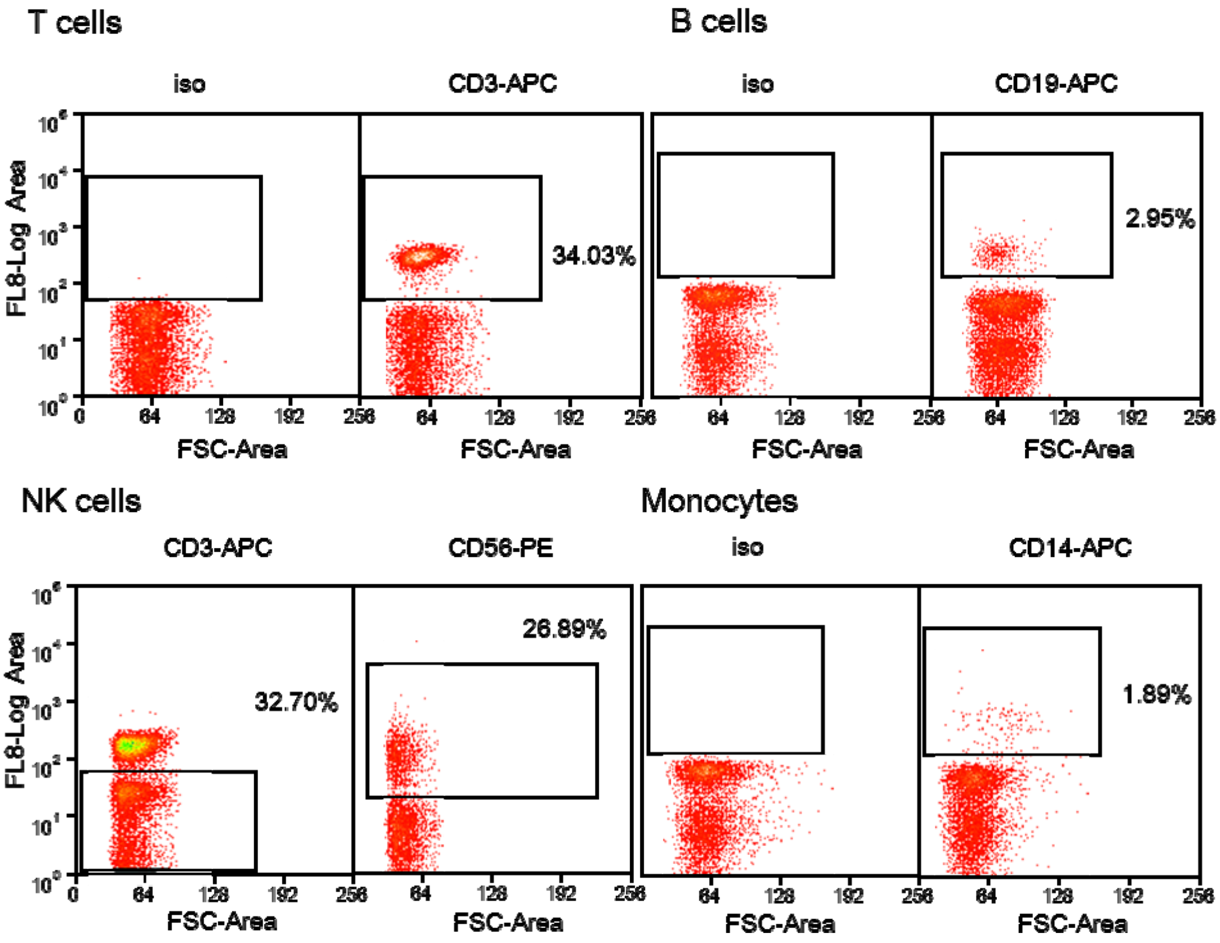

Supplementary Figure S5: The isolation gates that were set for the T cells, B cells, NK cells, and monocytes by flow cytometry sorter. Cells were stained with anti-CD3-APC, anti-CD19-APC, anti-CD56-PE, and anti-CD14-APC antibodies, followed by analysis on a Beckman MoFlo XDP Flow cytometer. Isotype controls with irrelevant specificities were included as negative controls. Human TruStain FcX™ (Fc Receptor Blocking Solution, Biolegend) was used to block Fc-receptors. T-lymphocyte cells (T cells); B-lymphocyte cells (B cells); NK (natural killer); PBMC (peripheral blood mononuclear cell); Isotype (iso).

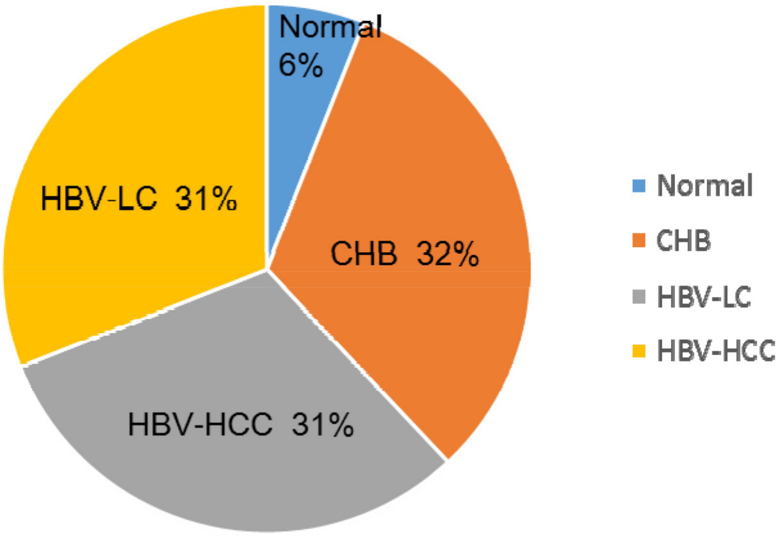

Supplementary Figure S6: The proportion of the samples for the clinical retrospective study. Chronic hepatitis B (CHB); hepatitis B virus-liver cirrhosis (HBV-LC); hepatitis B virus-HCC (HBV-HCC).

Supplementary Table 1

We performed clinical CGH array testing on 10 PBMCs samples from HBV-HCC patients from Eastern Hepatobiliary Surgery Hospital, Shanghai. The pathologic data of these 10 patients are summarized in supplementary table 1.

Supplementary Table S1: Pathology Data Form of 10 Patients with HBV-HCC

No.	sex	Age(yrs)	HBV-Ag	Child-Pugh	AFP(ug/L)	Tumor Size(cm)	Grade	Virus
1	female	45	+	A	24	6	III	B
2	male	39	+	A	453	5	III	B
3	male	67	+	A	12430	9	III	B
4	female	57	+	A	789	3	III	B
5	male	35	+	A	1340	6	IV	B
6	male	68	+	A	1490	11	III	B
7	male	54	+	A	430	4	II	B
8	male	43	+	A	12	3	III	B
9	male	42	+	A	7689	7	IV	B
10	male	51	+	A	530	4	III	B

*HBV-Ag; hepatitis B virus antigen; Child-Pugh score uses five clinical measures of liver disease, scored A to C, with A indicating the most severe condition. Grade, according to the Edmondson-Steiner grading system; B, hepatitis B virus; yrs, years.
